# A Word to Wives

**Published:** 1875-10

**Authors:** 


A Word to Wives.—No man ever truly prospered in the world without the co-operation of his wife. If she unite in mutual endeavors, or reward his labors with an endearing smile, with what confidence will he resort to his merchandise or his farm, fly over lands, sail over seas, meet difficulty and encounter danger, knowing that his labor will be rewarded by the sweets of home. Solicitude and disappointment enter the history of man's life; and he is but half provided for the voyage who finds but an associate for happy hours, while for the months of darkness and distress no sympathising partner is prepared.

---

The contented man is never poor; the discontented never rich.

